# Spintronic Bayesian Hardware Driven by Stochastic Magnetic Domain Wall Dynamics

**DOI:** 10.1002/advs.202520717

**Published:** 2026-03-17

**Authors:** Tianyi Wang, Bingqian Dai, Shijie Xu, Kin Wong, Yaochen Li, Yang Cheng, Qingyuan Shu, Haoran He, Puyang Huang, Hanshen Huang, Xixiang Zhang, Kang L. Wang

**Affiliations:** ^1^ Department of Electrical and Computer Engineering University of California Los Angeles California USA; ^2^ Physical Science and Engineering Division King Abdullah University of Science and Technology (KAUST) Thuwal Saudi Arabia

## Abstract

As AI expands into safety‐critical domains, reliability and uncertainty estimation have become essential, leading to the emergence of probabilistic computing. Conventional hardware platforms, such as CMOS, are inefficient for such implementations due to their deterministic nature, where the suppression of intrinsic fluctuations incurs high energy and computational costs to simulate probabilistic dynamics. At the core of this challenge is the absence of novel materials platforms and device architectures capable of natively supporting probabilistic behavior. To address this challenge, we present a Magnetic Probabilistic Computing (MPC) platform—an energy‐efficient, scalable hardware accelerator, where its inherent magnetic dynamics directly enable probabilistic computing. The MPC platform is based on Magnetic Tunnel Junction (MTJ) materials, which brings together three key mechanisms—thermally induced Domain Wall (DW) stochasticity, Voltage‐Controlled Magnetic Anisotropy (VCMA), and Tunneling Magnetoresistance (TMR)—to achieve fully electrical, tunable probabilistic functionality. As a representative demonstration, we implement a Bayesian Neural Network (BNN) inference structure and validate its functionality through CIFAR‐10 classification tasks by feeding experimentally acquired Gaussian distribution signals into a simulated BNN framework. Compared to standard 28 nm CMOS implementations, our approach achieves a seven‐orders‐of‐magnitude improvement in the overall figure of merit of the basic MPC unit, demonstrating substantial gains in area efficiency, energy consumption, and speed. These results highlight the promise of harnessing intrinsic spintronics material stochasticity within the MPC platform, opening new pathways toward reliable and trustworthy physical AI systems.

## Graphical abstract

Magnetic Probabilistic Computing (MPC) utilizes intrinsic stochastic dynamics in domain walls to establish a hardware foundation for uncertainty‐aware artificial intelligence. Thermally driven domain‐wall fluctuations, voltage‐controlled magnetic anisotropy, and TMR readout enable fully electrical, tunable probabilistic inference. A Bayesian neural network demonstration shows orders‐of‐magnitude gains in energy efficiency, area, and speed compared with conventional CMOS.

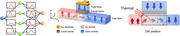

## 1 Introduction

The rapid advancement of artificial intelligence has led to its widespread deployment across diverse domains, rendering reliability and trustworthiness critical considerations [1, 2]. Conventional deep learning models, however, operate in a purely deterministic manner and lack inherent uncertainty awareness. Probabilistic Neural Networks (PNNs) have emerged as a compelling alternative [3, 4, 5, 6, 7], incorporating probability distributions to estimate prediction uncertainty. Yet, their practical implementation remains challenging due to high computational complexity, as probabilistic inference typically requires repeated sampling from posterior or intermediate distributions. Conventional hardware platforms, such as CMOS, are inefficient for such implementations due to their deterministic nature, where the suppression of intrinsic fluctuations incurs high energy and computational costs to simulate probabilistic dynamics. Addressing this limitation demands novel materials platforms and device architectures capable of natively supporting probabilistic behavior. Although various hardware‐based simulated solutions have been proposed to address this challenge [8, 9, 10, 11], very few experimental prototypes have been successfully demonstrated.

To address this challenge, we explore the spintronics materials and propose a physics‐driven computing strategy that harnesses emerging magnetic solitons—such as DWs [12, 13] and skyrmions [14, 15, 16, 17, 18, 19, 20, 21, 22, 23]—to exploit intrinsic magnetic dynamics [24, 25, 26], thereby establishing a new paradigm of physical platform tailored for probabilistic AI applications. Specifically, we introduce an MPC hardware that integrates DWs in MTJs [27, 28] with VCMA [29, 30], enabling the direct physical implementation of PNNs. The magnetic domains—potentially reaching nanoscale dimensions as small as 1 nm [31]—offer a compelling pathway for device scaling. The exceptionally low energy cost of DW motion, requiring only 27 aJ per operation [32], underscores its potential for ultra‐low‐power computation. DW motion speed can reach up to 4000 m/s [33], which supports high‐speed computing and memory operation. In addition, magnetic nonvolatility [34] enables seamless in‐memory computing, reducing data transfer overhead and enhancing system performance for complex neural workloads. Importantly, the stochastic nature of DW dynamics—combined with VCMA‐enabled tunable probability and TMR‐enabled high‐precision readout—makes this architecture inherently suitable for implementing probabilistic in‐memory computing. This architecture acts as a stochastic unit capable of generating output signals that follow a tunable Gaussian distribution. This enables efficient sampling and uncertainty estimation, offering a substantial improvement over conventional methods. As a representative demonstration, we implement a BNN, a representative class of PNNs, and validate its functionality through CIFAR‐10 classification tasks by feeding experimentally acquired Gaussian distribution signals into a simulated BNN framework. Benchmarking results show that, compared to conventional 28 nm CMOS implementations, our approach achieves a seven‐orders‐of‐magnitude improvement in the overall figure of merit, reflecting significant enhancements in area efficiency, energy consumption, and computational speed. This experimental demonstration underscores the potential of harnessing intrinsic spintronics material stochasticity in the MPC paradigm and opens new avenues toward the development of reliable and trustworthy physical AI systems.

## 2 Results

### 2.1 Magnetic Probabilistic Computing Platform and Physical Implementation of Tunable Probability

**FIGURE 1:**
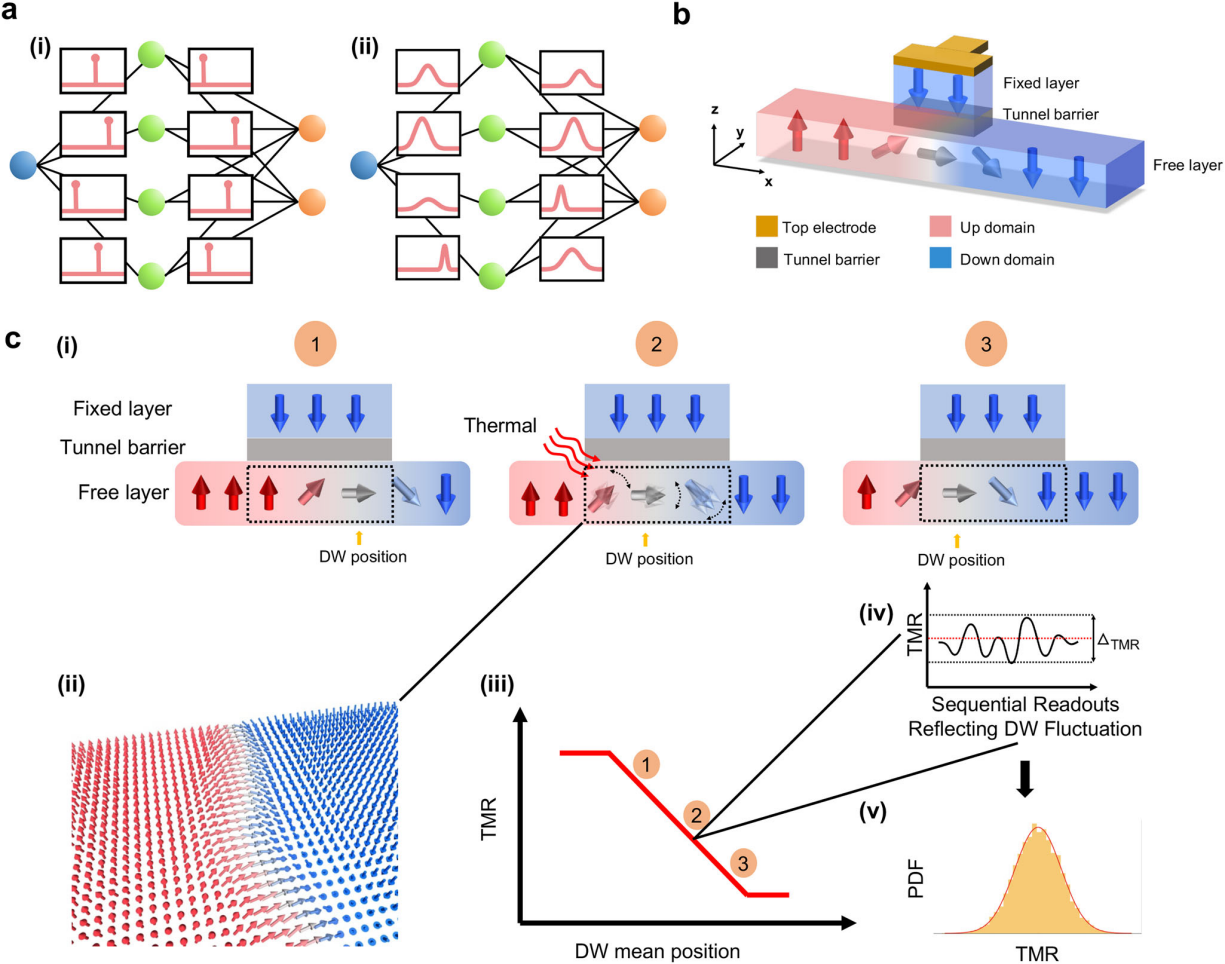
Magnetic probabilistic computing platform and physical implementation of tunable probability. (a) Structure of neural networks. (i) A point‐estimate neural network, where each weight is represented by a single value. (ii) Structure of a BNN, where each weight is represented by a trainable Gaussian distribution. (b) Schematic of the MPC device. The device consists of three layers: the free layer, the tunnel barrier layer, and the fixed layer. Red (blue) arrows indicate +z (‐z) magnetic domains. The grey arrow represents the in‐plane (+x) magnetization direction, corresponding to the magnetic DW. The top electrode, connected to the fixed layer, functions as the readout electrode. A reading voltage is applied across the MTJ stack to measure the TMR signal. (c) Operating mechanism of the device. (i) DWs located at distinct locations (labeled ①, ②, and ③) along the free layer. The dashed rectangle indicates the region probed by the TMR read head. Thermal excitations depicted by red wavy arrows perturb the magnetic moments, causing stochasticity in the DW position. (ii) Micromagnetic simulation illustrating DW snapshots. (iii) Each DW position corresponds to a specific point on the TMR versus DW mean position curve. (iv) When the DW mean position is held at a fixed location (e.g., Position ②), repeated TMR readouts exhibit stochastic fluctuations. (v) The resulting TMR signal variations follow a Gaussian distribution, as illustrated by the probability density function (PDF) plot.

BNNs extend conventional neural networks by representing weights as probability distributions rather than fixed‐point estimates [4, 5, 6]. In conventional neural networks, weights are simply deterministic and fixed values for each parameter, as depicted in Figure 1a (i). BNNs maintain a similar architectural framework but model the weights as probability distributions rather than point estimates, as illustrated in Figure 1a (ii). This probabilistic approach allows BNNs to quantify predictive uncertainty, thereby improving model robustness and reliability. While traditional neural networks optimize fixed weights by maximizing the likelihood function, BNNs leverage Bayes’ theorem to infer a posterior distribution over the weights given the observed training data.

Since the exact posterior *p*(*w*|*D*) is typically intractable due to the high dimensionality and complexity of neural network models, Variational Inference (VI) [35, 36, 37] is commonly employed. VI approximates the true posterior with a simpler, tractable distribution *q*(*w*; ϕ), often chosen to be Gaussian, where ϕ denotes the mean and standard deviation parameters. The objective is to minimize the Kullback–Leibler (KL) [38] divergence between the approximate and true posteriors. This is equivalent to maximizing the Evidence Lower Bound (ELBO) [39]. During training, weights are sampled from the variational distribution using the reparameterization method [40], enabling backpropagation. The model computes the expected log‐likelihood and KL divergence for each mini‐batch, and the ELBO is optimized through gradient‐based updates. This approach enables BNNs to learn both accurate predictions and meaningful uncertainty estimates. More details are discussed in Methods.

Based on the previous discussion, the implementation of a BNN requires generating Gaussian‐distributed weights with tunable mean and standard deviation to represent uncertainty in model parameters. In conventional CMOS‐based hardware, this process is nontrivial and introduces significant computational overhead. The standard approach involves multiple stages: generating base uniform random numbers, transforming these uniform values into Gaussian‐distributed samples, and finally applying scaling and shifting operations using digital multipliers and adders to control the desired mean and variance. Each of these steps incurs latency, energy consumption, and silicon area costs. As a result, implementing these processes in CMOS leads to large area footprints and elevated power consumption, especially when sampling must be performed repeatedly and in parallel across large neural networks. This complexity presents a major bottleneck for deploying BNNs on traditional hardware, particularly in edge AI applications where resources are limited and energy efficiency is critical. To address this challenge, we design a Magnetic Probabilistic Computing (MPC) platform—a hardware accelerator for energy‐efficient uncertainty‐aware computing based on intrinsic magnetic stochasticity. The hardware architecture is shown in Figure 1b. The proposed device is constructed on MTJ [27, 28] stacks comprising three layers: the free layer, the tunnel barrier, and the fixed layer. Within the free layer, the magnetic DW acts as the stochastic source of a Gaussian distribution. Red and blue arrows denote regions of positive and negative out‐of‐plane magnetization, respectively, while the grey arrow indicates the in‐plane magnetization direction, corresponding to the position of the DW. The top electrode, connected to the fixed magnetic layer, serves as the readout terminal. A voltage is applied vertically across the MTJ stack to measure the TMR signal. TMR arises from the spin‐dependent tunneling of electrons between two ferromagnetic layers separated by an insulating barrier. The resistance of the MTJ depends on the relative magnetization orientation of the free and fixed layers: it is low when the layers are aligned in a parallel (P) configuration and high when they are antiparallel (AP). Intermediate configurations, in which the free layer magnetization is only partially aligned, yield TMR values between the P and AP states. The TMR is proportional to the net magnetization beneath the readout terminal, enabling analog readout of the magnetic state. The TMR ratio is defined as:

$$TMR\;ratio=\frac{R_{AP}-R_{p}}{R_{p}}$$

where *R_AP_* and *R_p_* are the resistances in the AP and P states, respectively.

We next discuss how a Gaussian distribution can be realized based on DW dynamics. By precisely controlling the motion of the DW beneath the MTJ read terminal, the net magnetization in the sensing region can be finely tuned, enabling accurate modulation of the TMR signal. This controlled variability forms the basis for generating tunable Gaussian‐distributed outputs. Figure 1c (i) illustrates DWs positioned at distinct locations (labeled ①, ②, and ③) along the magnetic channel. Simultaneously, thermal excitations perturb the magnetic moment of the DW, causing it to fluctuate around its mean position. Figure 1c (ii) displays a DW profile obtained from finite temperature micromagnetic simulations using the MuMax3 software [41]. The irregular bending of DW showcases the effect of thermal fluctuations, serving as the physical basis of the observed stochasticity. Figure 1c (iii) provides an overview of the operating mechanism. The location of labels ①, ②, and ③ in Figure 1c (iii) presents the correspondence between the TMR signal and the DW's mean position in Figure 1c (i), enabling the control of the Gaussian mean. Simultaneously, thermal fluctuations alter the net magnetization around the mean value within the MTJ readout terminal, leading to variations in the TMR signal (see Figure 1c (iv)) and enabling the generation of Gaussian standard variation (Figure 1c (v)). To sum up, by combining the tunability of DW position and fluctuation, the Gaussian‐distributed random numbers are physically achieved. These principles will be experimentally verified in the next section.

### 2.2 Experimental Realization of the Magnetic Probabilistic Computing Device

**FIGURE 2:**
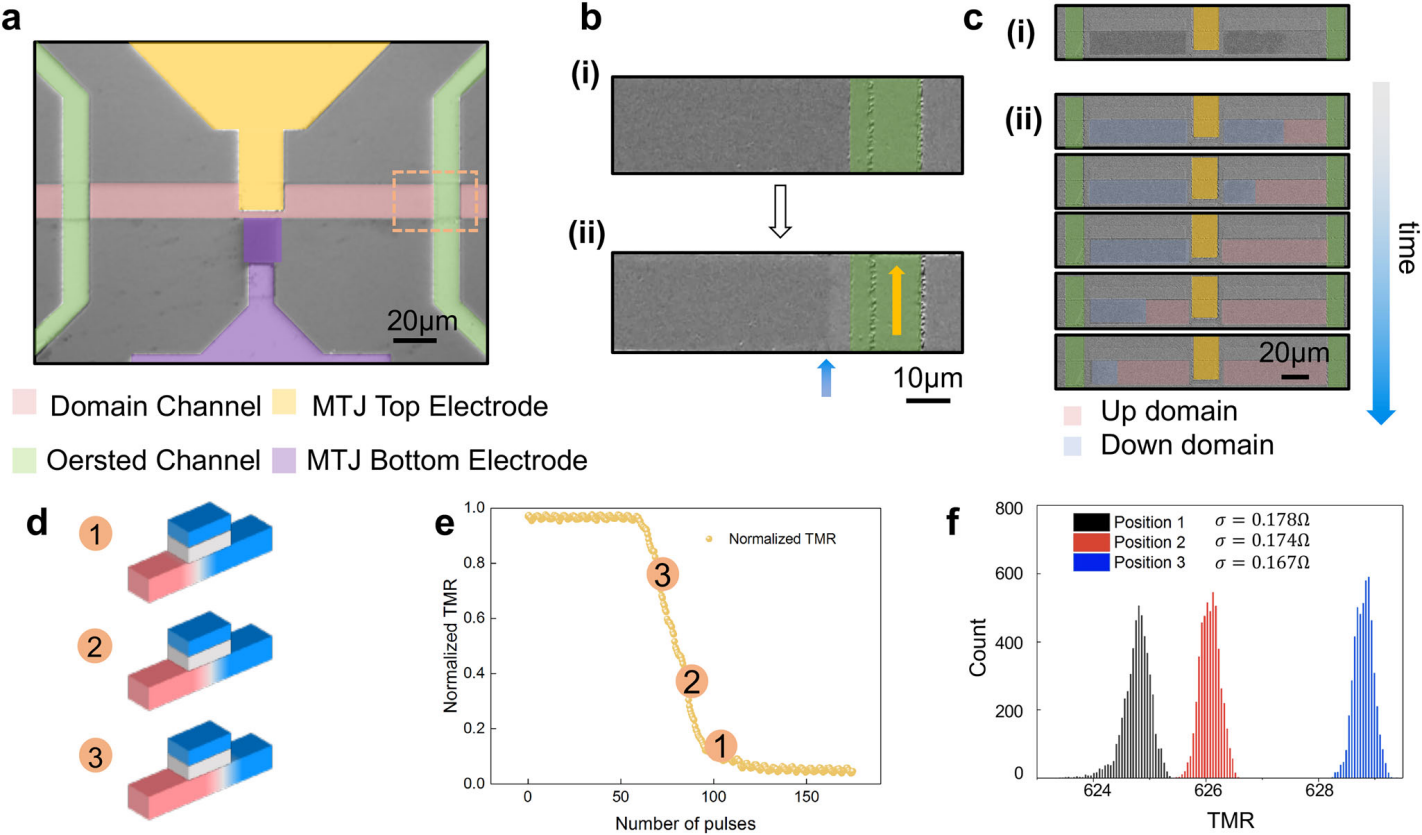
| Experimental realization of the magnetic probabilistic computing device. (a) Microscope image of a fabricated MPC device. (b) Experimental demonstration of DW creation: (i) Zoomed‐in image of the domain channel and Oersted channel, corresponding to the region within the orange dashed box in Figure 2a, (ii) MOKE image showing the DW formation after applying a current pulse to the Oersted channel, as indicated by the yellow arrow. The MOKE image contrasts regions of different magnetization: areas with magnetization pointing up (+M_Z_) appear white, while those with magnetization pointing down (−M_Z_) appear black. The boundary between these regions, marked by the blue arrow, indicates the position of the domain wall. (c) Experimental realization of DW motion in the MPC device characterized by MOKE: (i) Snapshot of DW motion. (ii) Sequence of images showing SOT‐driven DW motion from right to left. The up (down) domain region is shaded by red (blue) for better visualization. (d) Schematic illustration of the DW positioned at different locations beneath the MTJ. (e) Tuning of the Gaussian mean by DW position: TMR characterization of the MPC device as the DW propagates through the MTJ. Different TMR values correspond to different DW positions. The location of labels ①, ②, and ③ presents the correspondence between the TMR signal and the DW's position in Figure 2d, enabling the control of the Gaussian mean. (f) Experimentally obtained Gaussian distribution: Sampling of the Gaussian distribution TMR data with varying mean values and a fixed standard deviation. A total of 5,000 data points is collected for each DW position.

We next discuss the experimental implementations of our MPC platform. The experimental realization of the MPC device can be subdivided into three categories: (1) Generating a DW by injecting current into the Oersted channel. (2) Shifting the DW under the MTJ pillar by spin‐orbit torque (SOT). (3) Reading the TMR signal to generate a tunable Gaussian distribution. Figure 2a presents the optical microscope image of the MPC device after fabrication. The detailed materials stack development is discussed in Figure S1. The detailed device structure and fabrication process are discussed in Extended Data Figure 1 and Extended Data Figure 2. The domain generation is realized by injecting a current pulse into the right Oersted channel, which is marked in green in Figure 2a. The detailed zoomed‐in magneto‐optical Kerr effect (MOKE) image is shown in Figure 2b. Before the current injection, the magnetization within the domain channel is in the ‐z direction. After the current injection, a 6 µm domain is generated, and the DW location is marked by a blue arrow. DW shifting is achieved via SOT. When a charge current flows along the magnetic strip, it generates a transverse spin current through the spin Hall effect, which in turn exerts a torque on the DW, inducing its motion [42, 43]. A sequence of current pulses is applied to the domain channel to drive the DW from the right to the left. The DW motion process is captured by the MOKE images, which are presented in Figure 2c. Figure 2c (i) illustrates the DW motion at one specific time stamp. Figure 2c (ii) image series captures the whole DW motion process. Up‐domain and down‐domain regions are shaded with red and blue for better visualization. See Methods and Extended Data Figure 3 for details about DW generation and SOT‐driven DW motion. See Figures S2–S4 for DW stability test and reproducibility test.

Tuning a Gaussian distribution involves adjusting its mean and standard deviation. As discussed in the previous section, the mean value depends on the DW position under the MTJ pillar. When the DW resides at different positions beneath the MTJ pillar (Figure 2d), the TMR readout exhibits corresponding mean values. Figure 2e presents the TMR readout as the DW traverses beneath the MTJ pillar. The DW motion is driven by a series of current pulses applied sequentially, which incrementally shift the DW position along the domain channel. During this process, the TMR signal is continuously measured in real time. As the DW moves, the local magnetization beneath the readout head changes, leading to a gradual variation in the TMR value. The TMR value is recorded and plotted as a function of SOT pulse numbers. SOT pulse is set as 1mA‐0.1 ms. The TMR readouts at positions ①, ②, and ③ correspond to the respective DW locations shown in Figure 2d. If we stop the DW at a certain location, its mean position should be centered at the corresponding positions on the TMR loop. We then stop the DW at each of the three target positions shown in Figure 2d,e, and repeatedly collect 5,000 data points by continuously measuring the TMR signal over time. Due to thermal fluctuations, the DW exhibits random deviation around its mean position, resulting in corresponding fluctuations in the net magnetization beneath the readout head. This leads to stochastic variations in the TMR signal, i.e., the standard deviation of the Gaussian distribution. As these fluctuations are thermally driven, the resulting TMR values are expected to follow a Gaussian distribution centered at each target DW location. The results are shown in Figure 2f, where three distinct Gaussian distributions are observed, each with a different mean corresponding to the DW positions, but with identical standard deviations reflecting consistent thermal noise characteristics. The comparison between the ideal Gaussian and our experimentally obtained ones is presented in Extended Data Figure 4.

### 2.3 Voltage‐Controlled Standard Deviation of Gaussian Distribution

**FIGURE 3:**
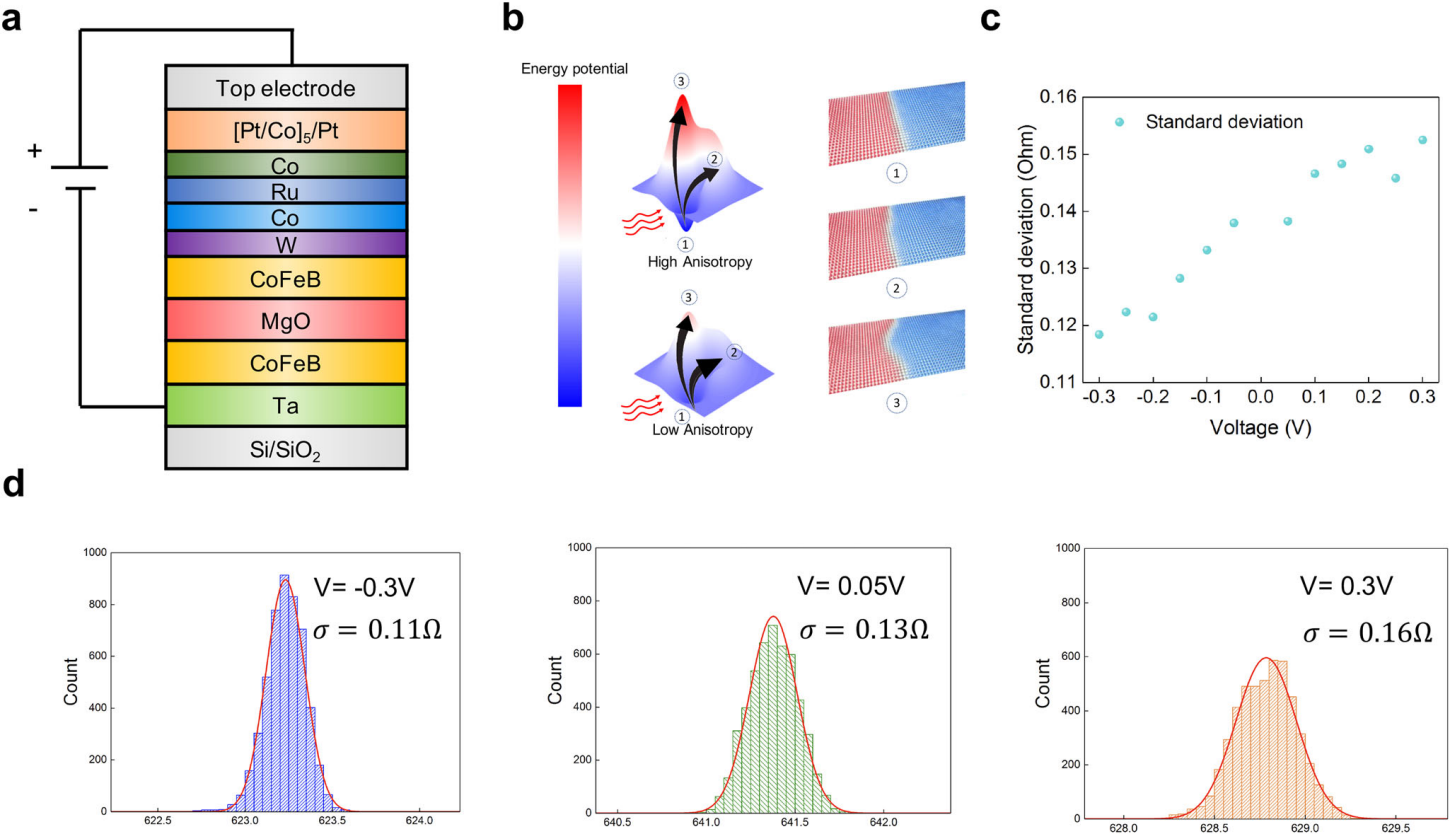
| Voltage‐controlled standard deviation of Gaussian distribution. (a) Materials stack. Applying a voltage across the MTJ modulates the perpendicular magnetic anisotropy (PMA): a positive (negative) voltage reduces (increases) the PMA. (b) Illustration of the DW energy landscape and corresponding DW configuration. Voltage‐controlled magnetic anisotropy (VCMA) modulates the PMA and reshapes the energy landscape. Under thermal excitation, DWs in a low‐PMA state are more likely to transition into an unstable (tilted) configuration due to a reduced energy barrier, leading to a larger standard deviation in the TMR readout. (c) Relationship between voltage and standard deviation. As the anisotropy changes, the DW's sensitivity to thermal fluctuations varies, resulting in different standard deviations in TMR. (d) Sampling of the Gaussian distribution TMR data with varying standard deviations. A total of 5,000 data points is collected for each voltage condition (V = ‐0.3 V, 0.05 V, 0.3 V).

Next, we investigate the tuning of the standard deviation of a Gaussian distribution through VCMA [29, 30]. VCMA enables the modulation of magnetic anisotropy energy in ferromagnetic materials via an applied electric field across an ultrathin insulating barrier. This electric field affects orbital hybridization at the ferromagnet/insulator interface, thereby modifying the perpendicular magnetic anisotropy (PMA), offering a promising mechanism for low‐power, high‐speed control of magnetic properties in spintronic devices. Figure 3a illustrates the application of voltage across the MTJ stack. This voltage modulates the PMA: a positive voltage reduces the PMA, whereas a negative voltage enhances it. As illustrated in Figure 3b, this modulation reshapes the energy landscape experienced by the DW. Micromagnetic simulation snapshots illustrate different DW configurations—straight (state ①) and tilted (states ② and ③)—corresponding to different positions in the energy landscape. In the high‐PMA regime, the energy barrier between the straight and tilted DW states is substantial, rendering thermal excitations insufficient to drive transitions between the two states. This causes the DW to mainly remain in the straight state, resulting in enhanced stability and a reduced standard deviation in the TMR readout. Conversely, when PMA is reduced, the energy barrier is lowered, enabling thermal fluctuations to more easily drive transitions between states. This increases the DW's sensitivity to thermal noise, leading to a larger standard deviation in TMR readout. This behavior is validated through micromagnetic simulations, as shown in Extended Data Figure 5. To experimentally confirm this effect, we center the mean position of the DW and use the same method used in Figure 2f to collect 5,000 data points under different voltage conditions. As anticipated, the standard deviation of the TMR signal varies with the applied voltage, following the predicted trend. Figure 3c quantifies the relationship between voltage and standard deviation, while Figure 3d displays sampled Gaussian distributions of TMR at *V_read_* =   − 0.3*V*,  0.05*V*,  0.3*V*.

### 2.4 Bayesian Neural Network Implementations by Magnetic Probabilistic Computing Platform

**FIGURE 4:**
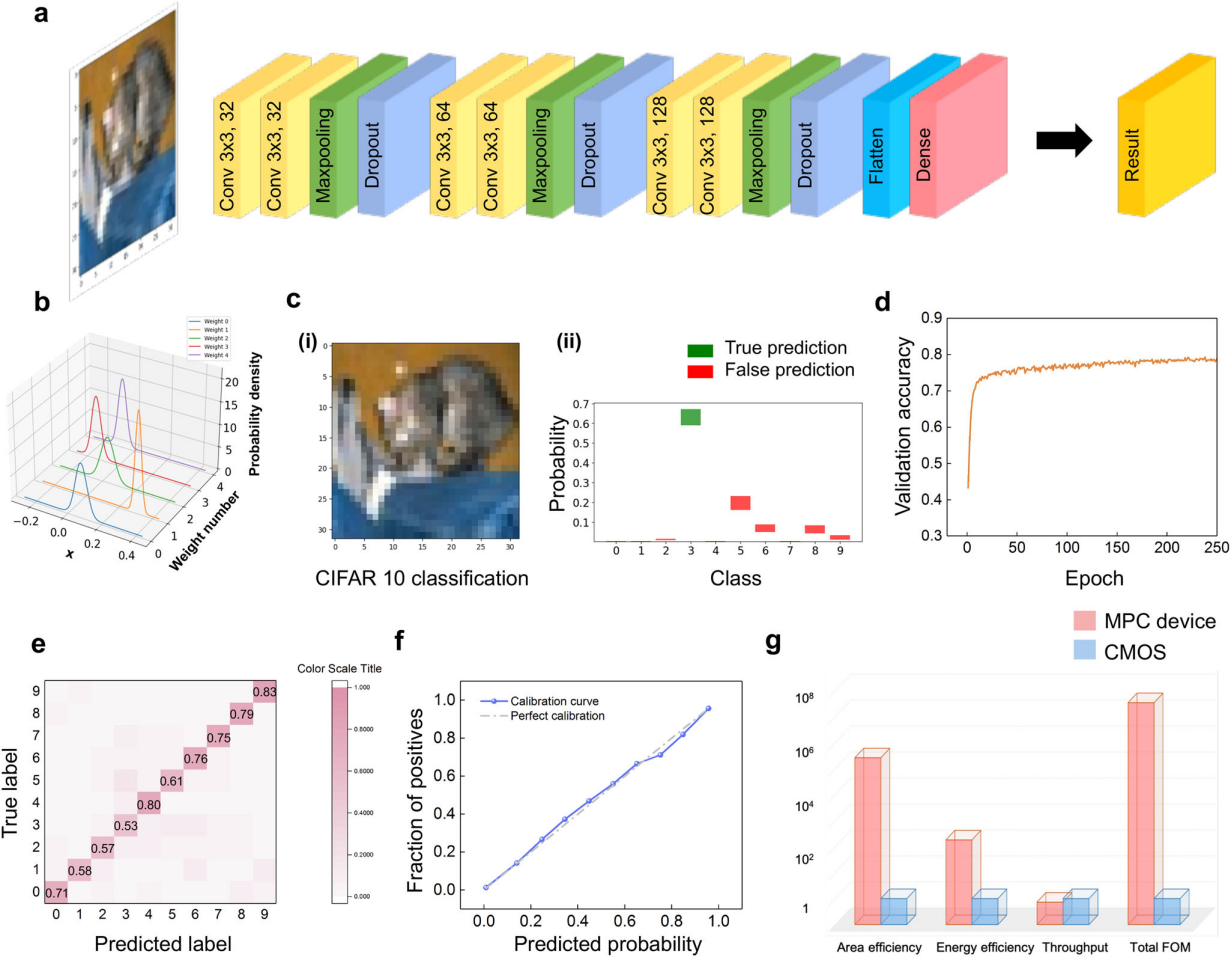
| Bayesian neural network implementations by magnetic probabilistic computing platform and benchmarking against CMOS. (a) BNN flow diagram. (b) Examples of Gaussian distributions in the first convolutional layer. (c) BNN prediction result. (i) CIFAR‐10 test image (cat, true label 3). (ii) BNN prediction on a CIFAR‐10 test image. The green bar indicates correct predictions, while the red bar indicates incorrect predictions. The width of the color bar represents the 95% confidence interval. (d) Validation accuracy vs. epochs. The final validation accuracy reaches 78.5% after 250 training epochs. (e) Confusion matrix. The y‐axis represents the true labels, while the x‐axis represents the predicted labels. The color scale indicates recognition probability, with the diagonal showing correct prediction rates. (f) Calibration curve. The curve closely aligns with perfect calibration, indicating neither overconfidence nor underconfidence. (g) Benchmarking between the MPC device and 28 nm CMOS technology on a logarithmic scale. MPC device achieves 7 orders of magnitude in FOM.

To demonstrate the practical applicability of our proposed platform, we integrated it into a complete BNN framework for inference tasks. Figure 4a shows the flow diagrams of the neural network, which consists of convolutional layers, maxpooling layers, dropout layers, flatten layers, and dense layers. The training of BNNs is finished in Google Colab [44] using TensorFlow Probability [45]. The detailed implementation is described in the Methods section. The first five weights from the convolutional layer are extracted and plotted in Figure 4b. As shown, these weights follow Gaussian distributions, each characterized by distinct mean values and standard deviations. We then map our Gaussian distributions from the MPC device to the ideal pre‐trained neural network weight to build our physical BNN (see Methods). Figure 4c (i) displays a test input image from the CIFAR‐10 dataset, corresponding to the class “cat” (label 3). The prediction results are shown in Figure 4c (ii), where green bars indicate correct predictions and red bars denote incorrect ones. The width of each bar represents the 95% confidence interval for the corresponding class. These results demonstrate that the proposed BNN not only performs classification but also provides meaningful uncertainty estimation. See the Uncertainty Quantification section in Supplementary Information for more details regarding quantifying aleatoric and epistemic uncertainties. To further calibrate the physical BNN, we validate the accuracy vs. epochs, and the final accuracy reaches 78.5% (Figure 4d), which is comparable with the result from state‐of‐the‐art [46]. We also conduct a recognition test and obtain the confusion matrix (Figure 4e), which indicates the recognition probability. The diagonal element corresponds to the correct prediction rate. To further calibrate the reliability of our physical BNN, we test and plot the reliability diagram in Figure 4f. The calibration curve aligns well with the perfect calibration, which indicates the neural network is neither overconfident nor underconfident.

We next present a benchmarking analysis comparing the performance of the MPC device to that of standard 28 nm CMOS technology, with a detailed discussion provided in the Supplementary Information. This benchmarking utilizes both directly measured experimental parameters and experimentally derived estimates to assess the device against standard CMOS across key performance metrics, including area efficiency, throughput, and energy consumption. The comparative results are summarized in Figure 4g.

First, we provide an overview of the standard approach by which CMOS circuits generate tunable Gaussian‐distributed random numbers for BNN implementations. In conventional CMOS architectures, this process typically involves three main steps: (1) generating base uniform random numbers, either through deterministic circuits such as linear feedback shift registers (LFSRs), which simulate randomness algorithmically, or through true random number generators (TRNGs) that exploit physical phenomena such as thermal noise, metastable behavior in flip‐flops, or jitter in ring oscillators to produce genuinely random outputs; (2) transforming these uniform random numbers into Gaussian‐distributed variables using mathematical techniques, most commonly the Central Limit Theorem (CLT) approach or the Box–Muller transformation; and (3) applying scaling and shifting operations to achieve the desired mean and variance, typically implemented with digital multiplier and adder circuits.

The benchmarking results reveal that our MPC device achieves a five‐orders‐of‐magnitude improvement in area efficiency and a three‐orders‐of‐magnitude improvement in energy efficiency compared to conventional CMOS implementations. This substantial advantage arises from the device's use of physical mechanisms—specifically, DW motion and VCMA—to achieve tunable Gaussian random number generation. In contrast, CMOS circuits require a large number of transistors to implement Box–Muller or CLT‐based transformations, along with additional scaling and shifting operations. The benchmarking analysis further shows that the throughput of the MPC device is in the same order as CMOS circuits, primarily constrained by the DW motion speed and the time required to initially position the DW. However, with further materials optimization, the DW velocity can be significantly improved [32, 33]. It is also important to note that the presented benchmarking is based on the time required to generate a single random number. In practical applications where N identical random numbers with the same distribution are needed (with N typically large), the time consumption in CMOS circuits scales approximately linearly with N. In contrast, in the MPC device, once the DW is set to the desired position, the only time per random number is the fast sampling time. The DW position setting time, which accounts for the majority of the time delay, becomes negligible over repeated sampling. As a result, the effective overall throughput of the MPC device can surpass that of conventional CMOS circuits in large‐scale applications.

According to the defined figure of merit (FOM), our device achieves an overall FOM improvement of seven orders of magnitude compared to conventional approaches from the basic unit level.

$$FOM=\frac{1}{Area\times Time\times Energy}$$

Meanwhile, it is important to emphasize that practical system‐level performance cannot be assessed solely based on improvements at the basic MPC computing unit level. In a complete BNN hardware implementation, overall performance is determined by how computational resources are distributed among Gaussian random number generation (RNG), matrix operations, and other processing blocks. The relative contribution of each component depends on the specific task, model size, and sampling strategy.

For the CIFAR‐10 image classification task specifically, our system‐level benchmarking shows that the MPC‐based BNN achieves approximately 25× lower energy consumption, comparable latency, and 5× smaller area compared with a conventional CMOS implementation. Details are discussed in Supporting Information. These results reflect realistic performance gains when the entire hardware architecture is taken into account, rather than only the underlying stochastic computing unit. Moreover, for more complex probabilistic models that require increased sampling and more frequent Gaussian RNG, the proportion of computation devoted to stochastic operations becomes higher. Under such conditions, the system‐level advantages of the MPC‐based approach are expected to become even more significant.

## 3 Conclusion

In conclusion, we present an MPC platform based on novel spintronic materials and device architectures that natively support probabilistic behavior. By integrating thermally induced DW stochasticity, VCMA, and TMR, the MPC platform enables efficient, device‐level sampling of tunable Gaussian‐distributed random numbers, paving the way for the practical deployment of physical probabilistic computing in AI applications. Through experimental validation on the CIFAR‐10 classification task, we demonstrate the feasibility of implementing BNNs using our MPC hardware, where experimentally measured Gaussian‐distributed signals are fed into a simulated BNN framework for system‐level evaluation. Compared to standard 28 nm CMOS implementations, our approach achieves a seven‐orders‐of‐magnitude improvement in the overall figure of merit, offering substantial gains in area, energy efficiency, and throughput from the basic device level. These results underscore the promise of leveraging intrinsic spintronics material stochasticity within the MPC platform for next‐generation trustworthy AI systems. While the achieved device‐level gains are significant, our current operation remains limited by several factors, particularly imperfections in the materials platform, which lead to DW pinning and highlight the need for further materials optimization and process refinement. Moreover, the present BNN implementation still operates in a hardware‐based simulation framework rather than a fully integrated on‐chip realization. Besides, the overall performance of BNN hardware is ultimately determined by the proportion of computation devoted to Gaussian random number generation relative to matrix operations and other supporting circuitry.

Looking ahead, the next step is to extend this platform toward integrated probabilistic processing units capable of executing entire stochastic inference pipelines in hardware. By scaling the MPC architecture into larger arrays and embedding it within compute‐in‐memory frameworks, more complex AI tasks can be supported, such as generative modeling. This advancement in materials platform and device design will further position spintronic probabilistic computing as a promising pathway toward the realization of reliable, trustworthy, and physically grounded AI systems.

## 4 Methods

### 4.1 Math Foundations of Bayesian Neural Network

In conventional neural networks, weights are optimized by simply maximizing the likelihood function, resulting in deterministic and fixed values for each parameter, as depicted in Figure 1a (i). BNNs maintain a similar architectural framework but model the weights as probability distributions rather than point estimates, as illustrated in Figure 1a (ii). The probabilistic feature enables BNNs to quantify uncertainty in predictions, thereby enhancing model robustness and reliability. The foundation of a BNN is rooted in Bayes’ theorem, which is expressed as:

$$p\;\left(w|D\right)=\frac{p\left(D|w\right)p\left(w\right)}{p\left(D\right)}$$

In this equation, *w* denotes the neural network weights. *p*(*w*) represents the prior distribution over the weights, capturing prior beliefs about their values before observing any data. *D* denotes the training dataset, and *p*(*D*) is the marginal likelihood, serving as a normalization constant. The term *p*(*D*|*w*) corresponds to the likelihood, indicating how well the model explains the observed data with weights *w*. *p*(*w*|*D*) is the posterior distribution, which reflects the updated beliefs about the weights after incorporating the information provided by the data. To make predictions for a new input x*, we need to integrate over all possible weights:

$$p\;\left(y^{*}|x^{*},D\right)=\int p\left(y^{*}|x^{*},w\right)\cdot p\left(w|D\right)\cdot dw$$

However, the posterior distribution *p*(*w*|*D*) is generally intractable in neural networks due to the high dimensionality and nonlinearity of the parameter space.

In practice, Variational Inference (VI) [35, 36, 37] is commonly utilized to approximate the posterior with a simpler, parameterized distribution. VI introduces an alternative distribution *q*(*w*; ϕ), selected to be computationally tractable, and aims to make it as close as possible to the true posterior. In this context, *q*(*w*; ϕ)is typically chosen from a family of Gaussian distributions, where the network weights are modeled as Gaussian random variables, and ϕ denotes the means and standard deviations of the distributions. The objective is then to optimize ϕ such that:

$$q\left(w;\phi \right)\approx p\left(w|D\right)$$

To quantify the difference between the selected Gaussian distribution and the true posterior, the KL divergence is introduced [38]. The KL divergence is a statistical measure that quantifies the discrepancy between a given probability distribution and a reference (or true) distribution. According to its definition, the goal is to minimize the following term:

$$KL(q\left(w;\phi \right)||p\left(w|D\right))=\int q\left(w;\phi \right)\ln\frac{q\left(w;\phi \right)}{p\left(w|D\right)}dw$$

Minimizing the KL divergence is equivalent to maximizing the ELBO [39], which serves as the final objective function to be optimized during the training of a BNN.

$$L\;\left(\phi \right)=E_{q\left(w;\phi \right)}\;\left[\ln p\left(D|w\right)\right]-KL\left(q\left(w;\phi \right)\left|\right|p\left(w\right)\right)$$

During training, weights are first sampled as Gaussian distributions using the reparameterization method [40]. For each mini‐batch, the network conducts a forward pass using sampled weights, evaluates the expected log‐likelihood, and computes the corresponding KL divergence. The ELBO is optimized by iteratively updating the parameters of the weight distributions. This training procedure enables the BNN to simultaneously learn accurate predictive behavior and effectively capture model uncertainty.

### 4.2 MOKE Microscopy Measurement

Polar magneto‐optic Kerr effect (MOKE) imaging was conducted using a custom‐built wide‐field MOKE microscope, offering a spatial resolution of 360 nm and a temporal resolution of 20 ms. This technique captures the magnetic signal along the out‐of‐plane (z) direction. An external magnetic field was applied using a GMW‐5201 Helmholtz coil, powered by a Kepco BOP 5–20D supply. To begin, a large negative out‐of‐plane magnetic field was applied to fully saturate the sample, after which a background image was acquired. Subsequent images were obtained by subtracting this background, thereby enhancing magnetic contrast.

### 4.3 Device Design and Fabrication

The detailed design of the MPC device is presented in Extended Data Figure 1. Extended Data Figure 1a illustrates the mask layout. Each device consists of SOT current channels (Channels 1 and 2), MTJ reading channels (Channels 3 and 4), and two pairs of Oersted current channels (Channels 5 and 6, as well as Channels 7 and 8). SOT current injected through Channels 1 and 2 drives the DW movement along the magnetic stripe. The TMR signal is detected through Channels 3 and 4, which are connected respectively to the top and bottom electrodes of the MTJ. DWs are initialized using Oersted currents applied either through Channels 5 and 6 or through Channels 7 and 8. Extended Data Figure 1b provides a zoomed‐in view of the region marked by the red rectangle in Extended Data Figure 1a. In this figure, the grey layer denotes the DW channel designed for DW propagation, the red layer represents the Ta contact layer that connects to the bottom electrode of the MTJ, and the black layer outlines the MTJ region. Extended Data Figure 1c depicts the fabricated device along with the typical measurement setup, where SOT current is injected into the bottom Ta layer, and the TMR signal is measured across the MTJ stack by applying a reading voltage. Extended Data Figure 1d,e illustrates cross‐sectional views of the fabricated device along the x–z and y–z planes, respectively. MPC devices are fabricated using standard photolithography, dry etching, wet etching, and evaporation techniques. For detailed fabrication steps, refer to Extended Data Figure 2.

### 4.4 Domain Generation, Domain Shifting, and TMR Measurement

To initialize the domain state shown in Figure 2, the MPC device was first saturated with a large negative out‐of‐plane magnetic field. Upon removal of the field, the device retained a negative out‐of‐plane magnetization. A current pulse generated by a Keithley 2636A Source‐Meter was applied to the Oersted field channel to generate a magnetic domain with +z orientation (Extended Data Figure 3a). The domain was then precisely repositioned to specific locations, enabling controlled targeting of various mean TMR values. Domain shifting was achieved through a sequence of SOT current pulses applied to the shifting channel using an Agilent 33250A arbitrary function generator (Extended Data Figure 3b). Subsequent TMR measurements were performed with the Keithley 2636A Source‐Meter. The typical experimental setup used for these measurements is illustrated in Extended Data Figure 1c, demonstrating how a voltage was applied across the MTJ stack. All described measurements were conducted at room temperature.

### 4.5 Gaussian Distribution Test

To verify that the data collected from the MPC device follows a Gaussian distribution, we utilized a quantile–quantile (Q–Q) plot [47] for validation and visualization. A Q–Q plot compares quantiles of the observed dataset with those from a theoretical Gaussian distribution. The procedure includes sorting experimental data in ascending order, calculating theoretical quantiles based on the Gaussian distribution corresponding to each data point, and plotting these sorted observed data quantiles (y‐axis) against the theoretical quantiles (x‐axis). If the observed data are normally distributed, the plotted points will align closely with the diagonal reference line. The ideal Gaussian Q–Q plot is illustrated in Extended Data Figure 4a, demonstrating the expected alignment when both sample and theoretical data follow a Gaussian distribution. The Q–Q plot generated from data measured with our MPC device is presented in Extended Data Figure 4b. Here, the experimental data points closely follow the reference line, confirming strong agreement with a Gaussian distribution.

### 4.6 Micromagnetic Simulation

Due to the absence of direct imaging of DW fluctuations in the MPC device, we performed Mumax3 micromagnetic simulations to qualitatively investigate DW dynamics under varying PMA conditions. We adjusted the anisotropy constant to simulate the VCMA effect. The grid size is set as 400 × 30 × 1 *nm* ^3^ and cell size is set as 2 × 2 × 1 *nm* ^3^. The following material parameters are chosen for the simulation: exchange constant *A_ex_* = 10^−11^ *J*/*m*, saturation magnetization *M_sat_* = 9 × 10^5^ *A*/*m*, out‐of‐plane uniaxial anisotropy, interfacial DMI 3 × 10^−3^ *J*/*m* ^2^, temperature *T*  =  20*K*, damping constant α  =  0.015. For low and high PMA cases, we use *K*_*u*1_ = 8 × 10^5^ *J*/*m* ^3^ and *K*_*u*2_ = 1.2 × 10^6^ *J*/*m* ^3^. The simulation result is shown in Extended Data Figure 5. Different PMA conditions lead to varying DW stability, resulting in different TMR readout variations.

### 4.7 BNN Training

To evaluate the performance of the BNNs based on our MPC platform, we carried out the following experimental steps. Figure 4a illustrates the architecture of the neural network, comprising convolutional layers, max‐pooling layers, dropout layers, flatten layers, and dense layers. Convolutional layers with configurations of 3 × 3 × 32, 3 × 3 × 64, and 3 × 3 × 128 are employed to extract image features such as edges and patterns. Max‐pooling layers with a 2 × 2 window size are used to emphasize image features while simultaneously reducing image dimensions to lower computational costs. Dropout layers with a rate of 0.25 mitigate overfitting, thereby improving the network's generalization capabilities. One flatten and two dense layers (with 128 and 10 neurons each) constitute the network's final processing stages.

The BNN training was conducted on the Google Colab platform using TensorFlow Probability, where the network was trained for 250 epochs with the ADAM optimizer. During training, each weight in the network was modeled as a Gaussian distribution characterized by a mean and standard deviation. After training, these learned statistical parameters were mapped to experimentally accessible values from our devices.

To enable physical implementation, each trained mean and standard deviation was discretized to match the quantized levels achievable by the magnetic probabilistic hardware. Specifically, the mean values were mapped to discrete TMR levels that correspond to the DW position beneath the MTJ read head. The standard deviation was mapped to the experimentally measured thermal fluctuation range of the DW position at each control state, which manifests as variability in the TMR output. This mapping ensures that each learned Gaussian weight distribution in the trained model is approximated by a corresponding physical distribution generated by the device.

In effect, the trained virtual network was translated into a physically realizable implementation, where both the central value and spread of each weight distribution are emulated by the stochastic behavior of the MPC system. The performance of this mapped experimental network was then evaluated using the test dataset. Key metrics such as average recognition accuracy, confusion matrix, and reliability analysis were assessed and reported in Figure 4.

### 4.8 Validation accuracy

It characterizes the overall predictive accuracy of the BNN on the validation dataset, assessing how well the model generalizes beyond training data. Our BNN reaches an overall accuracy of 78.5%, indicating a good prediction accuracy.

### 4.9 Confusion matrix

It provides a detailed breakdown of prediction performance by illustrating the distribution of true versus predicted classes. Specifically, it highlights the probability of classifying an input sample image into a particular category, facilitating a precise evaluation of classification accuracy and errors. The diagonal elements of the matrix represent correctly classified instances.

### 4.10 Calibration Curve

It evaluates the reliability of the uncertainty estimates by plotting predicted probabilities against observed frequencies. A well‐calibrated BNN yields a curve close to the ideal diagonal line, indicating accurate uncertainty quantification.

## Author Contributions

T.W., B.D., and S.X. designed, planned, and initiated studies. K.W. and Y.L. fabricated the devices. T.W. and B.D. conducted the MOKE and transport measurements and analyzed the data. T.W. performed the neural network training. K.L.W. supervised the project. T.W., B.D., S.X., and K.L.W. drafted the manuscript. All authors discussed the results and commented on the manuscript.

## Conflicts of Interest

The authors declare no conflicts of interest.

## Supporting information




**Supporting File 1**: advs74655‐sup‐0001‐SuppMat.docx.

**Supporting File 2**: advs74655‐sup‐0002‐SuppMat.docx.[Correction added on 24 March 2026, after first online publication: the Supporting Information file has been replaced in this version.]

## Data Availability

The data that support the findings of this study are available from the corresponding author upon reasonable request.
