# Supplementary material for: Spintronic Bayesian Hardware Driven by Stochastic Magnetic Domain Wall Dynamics
Source: Adv Sci (Weinh). 2026 Mar 17;13(27):e20717. doi: 10.1002/advs.202520717 (PMC13170188; doi:10.1002/advs.202520717)
Supplement: Supplementary file 1 — Supporting File 1: advs74655‐sup‐0001‐SuppMat.docx. [file ADVS-13-e20717-s002.docx]

**Supplementary Information of**

**Spintronic Bayesian Hardware Driven by Stochastic Magnetic Domain Wall Dynamics**

**Authors**

Tianyi Wang^1,*,†^, Bingqian Dai^1,*,†^, Shijie Xu^2,*^, Kin Wong^1^, Yaochen Li^1^, Yang Cheng^1^, Qingyuan Shu^1^, Haoran He^1^, Puyang Huang^1^, Hanshen Huang^1^, Xixiang Zhang^2^ and Kang L. Wang^1,†^

**Affiliations**

*^1^Department of Electrical and Computer Engineering, University of California, Los Angeles, California 90095, United States*

*^2^Physical Science and Engineering Division, King Abdullah University of Science and Technology (KAUST), Thuwal, Saudi Arabia.*

*These authors contributed equally to this work.

Corresponding author E-mail: ^†^tianyiwang0220@g.ucla.edu, ^†^bdai@g.ucla.edu, ^†^wang@ee.ucla.edu

**Fig. S1**

To balance DW stability and stochastic behavior, we performed extensive materials and device optimization to tune the PMA such that the DW remains sufficiently stable while retaining an appropriate level of fluctuation required for reliable stochastic sampling.

In the Ta/CoFeB/MgO material system, PMA is strongly dependent on the thickness of the CoFeB layer. By carefully tuning the **CoFeB thickness**, the PMA can be finely adjusted to reach an optimal regime in which the DW is relatively stable, while the thermal energy remains comparable to the effective PMA energy barrier. In this regime, thermal fluctuations can perturb the DW configuration without destroying its overall profile, thereby introducing the desired physical randomness. To experimentally identify this operating window, we fabricated devices based on materials with CoFeB thicknesses ranging from 0.92 nm to 1.04 nm, as shown in **Fig. S1(a)**. TMR measurements shows that the sample with 0.92 nm CoFeB exhibits high coercivity, indicating strong PMA(**Fig. S1(b)**); the 0.98 nm sample shows moderate coercivity(**Fig. S1(c)**); and the 1.04 nm sample exhibits an in-plane magnetic anisotropy trend (**Fig. S1(d)**). These results confirm that the PMA can be systematically tuned via CoFeB thickness engineering.

For our device operation, a relatively strong PMA is required to stabilize the DW, while thermal fluctuations must remain sufficiently active to serve as a source of intrinsic physical randomness. Based on this trade-off, we selected the material stack with a 0.98 nm CoFeB layer and performed all subsequent device measurements using this optimized configuration.

|  |
| --- |
| **Fig. S1 \| Materials stack and TMR loop. (a)** Materials stacks with different CoFeB thicknesses are fabricated into devices. TMR is thus measured based on these different stacks. **(b-d)** With the increasing of CoFeB thickness, the TMR loop shows strong PMA-normal PMA-in plane transition, indicating the fine control of materials properties. |

**Fig. S2**

To further verify DW stability, we provide complementary experimental evidence from both MOKE measurements and TMR measurements. As shown in **Fig. S2(a)**, the MOKE results indicate that the DW remains stable after its creation. An additional MOKE video is also provided to directly visualize the stable DW configuration over time. After driving the DW beneath the MTJ read head, we performed TMR measurements, as shown in **Fig. S2(b)**. The TMR signal remains stable, exhibiting thermal-induced fluctuations around a stable mean value.

Together, the MOKE and TMR measurements provide consistent and direct experimental evidence that by selecting an appropriate PMA, we achieve a balance between domain-wall stability and controlled thermal-induced stochasticity, which forms the physical basis of MPC.

|  |
| --- |
| **Fig. S2 \| Experimental results.** (a) MOKE imaging results showing the stable DW position. (b) TMR measurement results shows stable measurement results, indicating stable DW position. |

**Fig. S3**

To evaluate the reproducibility of the DW position, we performed additional experiments based on the setup shown in **Fig. S3(a)**. The TMR signal measures the net magnetization along the out-of-plane (𝑧) direction. As the DW position is tuned, the relative magnetic configuration beneath the MTJ changes, leading to a corresponding variation in the TMR readout. Therefore, the TMR signal directly reflects the DW position. In our experiments, fixed current pulse of 1 mA-1 ms was used. A series of pulses was applied to drive the DW toward and across the MTJ region. Once the DW entered the MTJ area, we recorded the corresponding normalized TMR values as a function of pulse number. This procedure was repeated 20 times under identical operating conditions.

The resulting normalized TMR versus pulse number curves are shown in **Fig. S3(b)**. For each pulse number, we calculated the mean TMR value and the associated standard deviation across the repeated measurements. These statistical results are summarized in **Fig. S3(c)**, where the TMR evolution is plotted with error bars representing the standard deviation. The small variation observed confirms that the DW position is highly reproducible across multiple repetitions under the same driving conditions.

We note that the reproducibility demonstrated here is based on our current device structure and experimental configuration. Further improvements are possible. From a device perspective, increasing the device dimensions can provide greater tolerance for DW positioning. From a materials perspective, reducing pinning sites through materials engineering can promote smoother and more uniform DW motion, thereby further enhancing positioning accuracy and reproducibility [1].

|  |
| --- |
| **Fig. S3 \| DW reproducibility test.** (a) Experimental setup of DW reproducibility test. (b) TMR vs pulse number relationships from different tests under same operation condition. (c) Statistical results of TMR vs pulse number over 20 repeated measurements. Mean values and standard deviations are calcuated to indicate the reproducibility of DW under repeated operation conditions. |

**Fig. S4**

We present the DW position tests based on TMR over the measurement periods. After creating the DW, we use SOT current pulses to drive the DW beneath the MTJ head and record the corresponding TMR signals. Data are acquired using a Keithley 2636 source meter at a sampling frequency of 60 kHz, with 5,000 data points collected for each measurement condition.

These data were collected when DW is at three different positions. Throughout the TMR measurement results in **Fig. S4 (a) to (c)**, no abrupt change or drift in the TMR signal was observed, indicating that the DW position remained stable during each measurement. These results are plotted together in **Fig. S4 (d)**, clearly showing that DW can be stabalized at different positions. It confirms that the DW mean position is indeed stationary while the TMR signal's stochasticity is being measured.

|  |
| --- |
| **Fig. S4 \| TMR results under different read voltages. (a)-(c)** TMR results measured when DW is at different positions. Stable signal confirms the stability of DW position. **(d)** Three test signals are plotted together, confirming different stable TMR mean values at various DW positions. |

**Uncertainty quantification**

The key advantage of a BNN is its ability to quantify predictive uncertainty, encompassing aleatoric (data) and epistemic (model) uncertainties. We further quantify aleatoric and epistemic uncertainties in BNN organized in the following sections.

1. Methods of quantifying aleatoric and epistemic uncertainties in BNN.
2. Simulation results of quantifying the aleatoric and epistemic uncertainties.

**Section 1. Methods of quantifying aleatoric and epistemic uncertainties in BNN**

Aleatoric uncertainty captures irreducible ambiguity inherent in the input data, arising from noise, class overlap, or limited observability. Epistemic uncertainty reflects uncertainty in the model parameters due to limited knowledge or distributional mismatch. To distinguish these different uncertainties, we need to apply different inputs, namely in-distribution (ID) inputs with noise and out-of-distribution (OOD) inputs without noise.

When noise is added to ID data, the model is still operating on familiar inputs and therefore exhibits low epistemic uncertainty. The increased uncertainty instead arises from ambiguity in the input itself, which broadens the conditional likelihood and manifests as higher aleatoric uncertainty that cannot be eliminated through additional training.

In contrast, OOD inputs lie outside the range of data the model has been trained on, where the learned posterior provides little guidance. As a result, different plausible model realizations can produce markedly different predictions, reflecting the model’s lack of experience with such inputs. This leads to high epistemic uncertainty, as the overall predictive uncertainty is driven primarily by disagreement between models rather than by noise or ambiguity in the data itself. This distinction explains why Bayesian neural networks typically exhibit increased aleatoric uncertainty for noisy ID data but a pronounced rise in epistemic uncertainty for OOD inputs, enabling a principled separation between data uncertainty and model uncertainty.

**Section 2. Simulation results of quantifying the aleatoric and epistemic uncertainties**

To further verify the capability of our MPC-based BNN, based on the main platform which has already been constructed in the manuscript, we create different inputs and conducted additional simulation in the following schemes:

1. Baseline: The original CIFAR-10 database images are used to analyze the baseline aleatoric and epistemic uncertainties in the ID case.
2. Additional simulation on OOD inputs: OOD inputs is defined to be a new category of inputs which don’t lie in any kind of previously trained classifications. At the same time, it must maintain the same or similar ambiguity as the other classifications. As a result, We adopt a leave-one-class-out protocol in which the model is trained on nine CIFAR-10 classes and the held-out class is treated as out-of-distribution during inference. In such case, the held-out class remains untrained to the model and gives the model (epistemic) uncertainty.
3. Additional simulation on ID inputs with additional noise. Images from the already trained classifications are added with additional noise. These noisy ID inputs is familiar to the model, but carries inherent noise which increases the ambiguity. This will give a elevated aleatoric uncertainty.

The uncertainties is evaluated using the entropy based uncertainties.

For a given input *x*, the Bayesian model gives *s* stochastic predictions.

$$p^{\left( s \right)}\left( y | x \right)=\left( {p_{1}}^{\left( s \right)},{p_{2}}^{\left( s \right)},\ldots{p_{9}}^{\left( s \right)} \right), s=1,2,\ldots S$$

Each $p^{\left( s \right)}$ is a probability vector.

To get the predictive distribution by averaging

$$\overline{p}\left( y | x \right)=\frac{1}{S}\sum_{s=1}^{S} p^{\left( s \right)}\left( y | x \right)$$

Component wise:

$$\bar{p}_{c}=\frac{1}{S}\sum_{s=1}^{S} {p_{c}}^{\left( s \right)}$$

**Total uncertainty:**

The total uncertainty in the classification task is entropy, which is computed as:

$$U_{pred}\left( x \right)=H\left( \bar{p} \right)=-\sum_{c=1}^{C} \bar{p}_{c}log\bar{p}_{c}$$

**Aleatoric uncertainty:**

The entropy per single run inference is defined as

$$H^{\left( s \right)}\left( x \right)=-\sum_{c=1}^{C} {p_{c}}^{\left( s \right)}log{p_{c}}^{\left( s \right)}$$

Averaging over samples give the aleatoric uncertainty

$$U_{aleotoric}\left( x \right)=\frac{1}{S}\sum_{s=1}^{S} H^{\left( s \right)}\left( x \right)$$

**Epistemic uncertainty:**

Epistemic uncertainty is computed as

$$U_{epistemic}\left( x \right)={U_{pred}\left( x \right)-U}_{aleotoric}\left( x \right)$$

Based on the aforementioned inputs and evaluation standards, we summarized the results below.

| Inputs | Predicted uncertainty | Aleatoric uncertainty | Epistemic uncertainty |
| --- | --- | --- | --- |
| Baseline | 1.1028 | 0.9210 | 0.1818 |
| ID with noise | 1.2385 | 1.0346 | 0.2039 |
| OOD | 1.6613 | 1.4419 | 0.2193 |
| **Table. S1 \| Uncertainties characterization.** | | | |

For ID data, both aleatoric and epistemic uncertainties remain low, indicating confident and well-calibrated predictions. When noise is added to ID inputs, the expected uncertainty increases, reflecting elevated aleatoric uncertainty induced by input corruption, while the epistemic uncertainty shows only a modest change. In comparison, OOD inputs exhibit higher predictive uncertainty together with a moderate and systematic increase in epistemic uncertainty, consistent with increased model uncertainty when encountering samples outside the training distribution. These results indicate that the BNN is able to distinguish and appropriately respond to different sources of uncertainty.

**Benchmarking between MPC and 28nm CMOS**

We present a detailed area, throughput, and energy analysis of our magnetic probabilistic computing (MPC) device versus standard 28nm CMOS. This benchmarking utilizes both directly measured experimental parameters and experimentally derived estimates to assess the device against standard CMOS across key performance metrics, including area efficiency, throughput, and energy consumption. The comparative results are summarized in Main text Figure. 4g.

**Estimation for magnetic probabilistic computing device**

The area of the BNN MTJ device is primarily constrained by fabrication limits and the stability of magnetic domain wall. The MTJ width is set by the fabrication resolution, which in this work is assumed to be 30 nm according to TSMC 28nm technology node. The device length is determined by the accuracy required for generating Gaussian random numbers. To achieve 8-bit Gaussian random number generation, precise control over domain wall displacement is necessary. Previous studies have demonstrated sub-nanometer tunability of domain wall positions [2]; here, we conservatively assume a tunable step size of 1 nm. This yields an active MTJ pillar length of 256 nm (corresponding to 2^8^ steps). Additionally, to account for domain wall nucleation via the Oersted field, we include an extra 75 nm region for domain wall generation and stabilization. Therefore, the total device area is calculated as

$$Area=W_{MTJ}\times(L_{MTJ}+75nm)=30nm\times\left( 256nm+75nm \right)=9.93\times{10}^{-3}{\mu m}^{2}$$

The total time latency arises from two main contributions: the domain wall motion time and the sampling time. Here we ignore the time needed for Oersted field-driven domain wall generation because such process is only required during the initialization. The domain wall motion time is governed by the domain wall velocity, reported in prior work to be approximately 25 m/s [3]. Accordingly, the time required to position the domain wall under the MTJ read head is estimated as:

$$t_{DWM}=\frac{L_{MTJ}}{v_{DW}}=\frac{256nm}{25m/s}=10.24ns$$

The sampling time is determined by the RC time constant of the readout circuit, where we assume an MTJ resistance of 100 kΩ and a typical sampling capacitance of 1 fF, yielding:

$$\tau=R_{MTJ}\times C_{sample}=0.1ns$$

Thus, the total estimated time latency per operation is:

$$t_{total}=t_{read}+t_{DWM}=10.34ns$$

The calculated latency corresponds to the time required to generate a single Gaussian random number. However, when sampling a large number of Gaussian random numbers, the domain wall can be held at the mean position, and only the sampling time contributes to the overall latency. This significantly reduces the effective time delay per sample.

The total energy consumption consists primarily of two components: the energy required for domain wall shifting and the energy required for readout. To estimate the domain wall shifting energy, we use the following experimental parameters: a current density $J_{SOT}=1.1\times{10}^{12}A/m^{2}$, Resistivity $\rho=9.4\times{10}^{-7} m\cdot\Omega$. Shift current can thus be calculated.

$$I_{shift}=J_{SOT}\times thickness\times W_{MTJ}=1.65\times{10}^{-4}A$$

The resistance of the strip can be calculated as

$$R_{strip}=\rho\times\frac{L_{MTJ}+75nm}{W_{MTJ}\times thickness}=2.07k\Omega$$

The shift energy can be calculated as

$$E_{shift}={I_{shift}}^{2}\times R_{strip}\times t_{DWM}=5.77\times{10}^{-13}J$$

We take the $V_{read}$ to be 1V, then the reading energy can be calculated as

$$E_{read}=\frac{{V_{read}}^{2}}{R_{MTJ}}\times t_{read}=1\times{10}^{-15}J$$

The energy for charging the sampling capacitors can be calculated as

$$E_{cap}={C_{sample}V_{read}}^{2}=1\times{10}^{-15}J$$

In the MPC implementation, a LUT is required to map the readout voltage and SOT driving conditions to the corresponding Gaussian mean (μ) and standard deviation (σ). To estimate the energy cost associated with LUT access, we adopt values reported in prior work. Specifically, the energy consumption of a standard CMOS LUT is reported to be 1.22 pJ in 130 nm technology [4]. Using standard technology scaling, this corresponds to an estimated energy cost of approximately 0.25 pJ at the 28 nm node.

The total energy is

$$E_{total}=E_{shift}+E_{read}+E_{cap}=0.829pJ$$

The parameters for estimation are listed in the table.

| Parameters | Definition | Value |
| --- | --- | --- |
| $v_{DW}$ | Domain wall max velocity | $25m/s$ |
| $\rho$ | Strip resistivity | $9.4\times{10}^{-7} m\cdot\Omega$ |
| $J_{SOT}$ | Shift current density | $1.1\times{10}^{12} A/m^{2}$ |
| $thickness$ | Strip thickness | $5 nm$ |
| $R_{MTJ}$ | Resistance of MTJ | $100 k\Omega$ |
| $L_{MTJ}$ | Length of MTJ | $256 nm$ |
| $W_{MTJ}$ | Width of MTJ pillar | $30 nm$ |
| $C_{sample}$ | Sample capacitor | $1 fF$ |
| **Table. S2 \| Parameters for estimation.** | | |

**Estimation for CMOS circuits**

The ability to generate Gaussian-distributed random numbers with tunable mean and variance is critical in the Bayesian neural network implementation. In CMOS circuits, this process typically involves three major steps: (1) generating base uniform random numbers, (2) transforming these uniform numbers into Gaussian-distributed variables through mathematical techniques, (3) applying scaling and shifting operations to achieve the desired mean and variance. This section systematically describes the standard methods employed in each step to realize tunable Gaussian distributions in digital CMOS implementations.

Step 1: Several methods exist for generating uniformly distributed random numbers in CMOS circuits. Deterministic circuits, such as Linear Feedback Shift Registers (LFSRs), simulate randomness through algorithmic means. LFSRs, typically constructed from flip-flops and XOR gates, produce sequences that appear random but are in fact deterministic, categorizing them as pseudo-random number generators (PRNGs). In addition to deterministic approaches, true random number generators (TRNGs) leverage physical sources of randomness. TRNG circuits utilize phenomena such as thermal noise, metastable behavior in flip-flops, or jitter in ring oscillators to produce genuinely random outputs. These methods can create uniformly distributed random variables for further use.

Step 2: Two primary methods are commonly employed for generating Gaussian-distributed random variables: the Central Limit Theorem (CLT) and the Box–Muller transformation. The CLT states that the normalized sum (or mean) of a sufficiently large number of independent and identically distributed random variables converges to a standard normal distribution. However, due to its inherent reliance on sampling, the CLT-based method may exhibit poor accuracy in the distribution tails when the sample size is limited. To avoid such limitations and ensure high fidelity in the generated distribution, Box–Muller transformation is often adopted, which produces truly Gaussian-distributed random variables without requiring extensive sampling.

The Box–Muller transform, originally proposed by George Edward Pelham Box and Mervin Edgar Muller, is a mathematical technique for generating pairs of independent, standard normally distributed random numbers from uniformly distributed random numbers. Given two independent samples $x_{1}$​ and $x_{2}$ drawn from a uniform distribution over the interval $\left( 0, 1 \right)$, the transformation is defined as follows:

$$z_{1}=\sqrt{-2\ln x_{1}}\cos\left( 2\pi x_{2} \right)$$

$$z_{2}=\sqrt{-2\ln x_{1}}\sin\left( 2\pi x_{2} \right)$$

Two samples $x_{1}$ and $x_{2}$ drawn from the uniform distribution over the interval $\left( 0, 1 \right)$, are transformed into $z_{1}$​ and $z_{2}$​, two independent standard normal random variables with zero mean and unit variance.

Step 3: In digital CMOS circuits, once a Gaussian-distributed random variable $z_{0}$ with zero mean and unit variance is generated, it is scaled to a target mean μ and standard deviation σ through a simple linear transformation:

$$z_{scaled}=\sigma z_{0}+\mu$$

This scaling operation is typically implemented using digital arithmetic blocks. The standard normal variable is first multiplied by the desired standard deviation using a digital multiplier. The resulting scaled value is then offset by the target mean through a digital adder circuit, producing the final Gaussian-distributed output.

***Energy cost:***

CMOS-based random number generators typically consume approximately 2 pJ per generated bit. For instance, a digital TRNG implemented in CMOS was reported to achieve an energy consumption of 2.58 pJ per bit [5]. To further generate Gaussian-distributed random numbers from uniform inputs, Box–Muller transformation involves several computational stages: logarithm calculation, square root calculation, trigonometric functions, and multiplications. Logarithm and square root functions, typically realized using lookup tables or iterative methods, are computationally intensive and can consume approximately 100–200 pJ per operation. Trigonometric functions are often implemented using CORDIC algorithms, requiring 10–20 pJ per sine or cosine evaluation. [6, 7] Multiplication, when performed using an 8×8-bit multiplier in 28nm CMOS, generally consumes around 2 pJ per operation [8]. To scale the output to arbitrary mean and variance, the final stage includes an additional 8-bit multiplication and addition. This step adds approximately 3 pJ per sample, based on typical energy costs for multipliers (∼2 pJ) and adders (<1 pJ).

Considering all stages—including uniform RNG generation, Box–Muller transformation, and final scaling—the total energy required to generate a single 8-bit Gaussian random number in 28nm CMOS is estimated to be in the range of **119–229 pJ.**

***Time latency:***

Uniform random number generators (RNGs), typically implemented using linear feedback shift registers (LFSRs) or similar pseudo-random number circuits can produce output within **1–2 ns**. In prior work, a latency of **2.65 ns** has been reported for a CMOS RNG [9]. The latency for computing the Box Muller transformation depends heavily on the underlying implementation. If lookup tables (LUTs) are used, these functions can be completed in a single clock cycle. In contrast, iterative or CORDIC-based implementations typically require multiple clock cycles [10], with reported latencies ranging from **1 to 10 cycles** depending on pipeline depth, target precision, and clock speed. The multipliers used in the Box–Muller stage are generally fully combinational and optimized for a single pipeline stage. As such, these operations typically execute within a **single-cycle latency** in pipelined digital processors. The final scaling stage, which adjusts the Gaussian sample to match a target mean and variance, involves one additional 8-bit multiplier and one adder. This stage typically introduces an additional **1–2 ns** of delay.

Considering all functional stages, the **total latency** to generate one 8-bit Gaussian-distributed random number in 28nm CMOS is estimated to be approximately **8–15 ns**.

***Area efficiency:***

The pseudo-random number generator is most commonly realized using an 8-bit linear feedback shift register (LFSR) [11]. D flip-flops, XOR gates, and control logic require approximately 60 gate equivalents (GE), translating to around 90–120 µm² in TSMC 28nm standard-cell libraries. For the Box–Muller transformation, both the natural logarithm and square root functions can be implemented using small lookup tables (LUTs) or iterative logic, each requiring roughly 250–300 GE. Trigonometric operations such as sine and cosine are typically implemented using CORDIC algorithms [10], which require additional shift-and-add stages and control logic. These blocks can consume about 500–1000 µm² in total. Finally, to support scaling to arbitrary mean and standard deviation, the design includes an additional multiplier and an adder, contributing another 1000 µm².[12]

The total area required to implement a Gaussian random number generator in 28nm CMOS is approximately **0.0026 to 0.0032 mm^2^**.

|  | Energy (pJ) | Time latency (ns) | Area(μm^2^) |
| --- | --- | --- | --- |
| Uniform RNG | 2 | 1-2 | 90-120 |
| Box-Muller | 115-225 | 7-11 | 800-2300 |
| Scaling | 3 | 1-2 | 1000 |
| Total | 119-229 | 8-15 | 2600-3200 |
| **Table. S3 \| Benchmarking results for 28nm CMOS.** | | | |

**Benchmarking between different neural network implementations**

Unlike deterministic neural network (DNNs), which perform a single forward pass to produce a point estimate, BNNs require Monte Carlo sampling over multiple weights to generate probabilistic outputs and quantify uncertainty. This inevitably introduces additional computational overhead and inference latency, representing an intrinsic trade-off of BNN rather than an hardware implementation inefficiency. Therefore, a fair benchmarking should account for both the added computational cost and the information gain provided by uncertainty estimation.

To guarantee a meaningful and appropriate comparison, we adopt the following benchmarking protocol. We benchmark the MPC-enabled BNN against a deterministic CMOS DNN. In addition, we include a CMOS-based BNN as a more appropriate baseline for fair comparison. For both the MPC BNN and CMOS BNN, classifications are aggregated over 10 single runs of stochastic inference to estimate the predictive mean and uncertainty. Based on the BNN architecture described in the main text, we assume a total of 1 million network parameters. Furthermore, we account for 10,000 random number generation units in both the MPC and CMOS implementations.

Under these assumptions, we compare the different neural network implementations in terms of uncertainty estimation capability, energy consumption, inference latency, and area. Key results are summarized in **Table S4**. Both the MPC-based and CMOS-based BNNs provide explicit uncertainty characterization. In contrast, while the DNN [13] exhibits lower energy consumption and faster single-pass inference, it fundamentally lacks uncertainty estimation due to the deterministic nature of its architecture. Hence, a more appropriate comparison is therefore between the MPC BNN and the CMOS BNN. In this case, our results show that the MPC implementation achieves significantly improved energy and area efficiency while maintaining comparable inference latency. This significant improvement arises from the physical source of randomness in our energy-efficient, scalable MPC device, where intrinsic magnetic dynamics directly enable probabilistic computing. We note that this benchmarking is based on the specific CIFAR-10 network architecture considered in this work; for more complex models requiring increased Gaussian random number generation and sampling, the advantages of the MPC-based approach are expected to become even more pronounced.

| **Types of Neural Network** | **Uncertainty characterization** | **Energy cost** | | | **Latency** | | | **Area** | **Accuracy** |
| --- | --- | --- | --- | --- | --- | --- | --- | --- | --- |
|  |  | **Sampling** | **Single run** | **Classification** | **Sampling** | **Single run** | **Classification** |  |  |
| MPC BNN | Yes | 0.829 pJ | 4.629 $\mu$J | 46.29 $\mu$J | 10.34 ns | 14.34 ms | 143.4 ms | ~6 ${mm}^{2}$ | 78.5% |
| CMOS DNN^[13]^ | No | N/A | N/A | 3.8 $\mu$J | N/A | N/A | 4 ms | 6 ${mm}^{2}$ | ~80% |
| CMOS BNN | Yes | 119 pJ | 122.8 $\mu$J | 1228 $\mu$J | 8 ns | 12 ms | 120 ms | 32 ${mm}^{2}$ | ~80% |
| **Table. S4 \| Benchmarking results.** Benchmarking results of MPC-BNN, CMOS-DNN, and CMOS-BNN. DNN doesn’t include sampling, so it only shows the basic classification energy cost and latency. On the other hand, MPC BNN and CMOS BNN includes additional sampling energy and latency, which are collected from Supplementary Information and added over the basic classification. All neural network platforms perform the same CIFAR-10 tasks. | | | | | | | | | |

Overall, our benchmarking highlights the fundamental differences and intrinsic trade-off between DNN and BNN. The proposed MPC-enabled BNN is not intended to replace DNN in all scenarios, but rather to serve as a complementary solution in applications where uncertainty awareness and robustness are critical. In such cases, our MPC-based implementation demonstrates a clear advantage over conventional CMOS designs.

**References**

[1] Kumar, D., Chung, H.J., Chan, J., Jin, T., Lim, S.T., Parkin, S.S., Sbiaa, R. and Piramanayagam, S.N., 2023. Ultralow energy domain wall device for spin-based neuromorphic computing. ACS nano, 17(7), pp.6261-6274.

[2] Spethmann, Jonas, et al. "Zero-field skyrmionic states and in-field edge-skyrmions induced by boundary tuning." Communications Physics 5.1 (2022): 19.

[3] Emori, S., Bauer, U., Ahn, SM. et al. Current-driven dynamics of chiral ferromagnetic domain walls. Nature Mater 12, 611–616 (2013).

[4]D. Brito, T. G. Rabuske, J. R. Fernandes, P. Flores and J. Monteiro, "Quaternary Logic Lookup Table in Standard CMOS," in IEEE Transactions on Very Large Scale Integration (VLSI) Systems, vol. 23, no. 2, pp. 306-316, Feb. 2015

[5] Pamula, V.R., Sun, X., Kim, S.M., ur Rahman, F., Zhang, B. and Sathe, V.S., 2019. A 65-nm CMOS 3.2-to-86 Mb/s 2.58 pJ/bit highly digital true-random-number generator with integrated de-correlation and bias correction. IEEE Solid-State Circuits Letters, 1(12), pp.237-240.

[6] Wang, Y., Deng, D., Liu, L., Wei, S. and Yin, S., 2021, June. Lpe: Logarithm posit processing element for energy-efficient edge-device training. In 2021 IEEE 3rd International Conference on Artificial Intelligence Circuits and Systems (AICAS) (pp. 1-4). IEEE.

[7] Wu, D., Chen, T., Chen, C., Ahia, O., San Miguel, J., Lipasti, M. and Kim, Y., 2019, July. SECO: A scalable accuracy approximate exponential function via cross-layer optimization. In 2019 IEEE/ACM International Symposium on Low Power Electronics and Design (ISLPED) (pp. 1-6). IEEE.

[8] Aguirre-Hernandez, M. and Linares-Aranda, M., 2008, November. Energy-efficient high-speed CMOS pipelined multiplier. In 2008 5th International Conference on Electrical Engineering, Computing Science and Automatic Control (pp. 460-464). IEEE.

[9] Osama, M., Gaber, L. and Hussein, A., 2016, February. Design of high performance Pseudorandom Clock Generator for compressive sampling applications. In 2016 33rd National Radio Science Conference (NRSC) (pp. 257-265). IEEE.

[10] Andraka, R., 1998, March. A survey of CORDIC algorithms for FPGA based computers. In Proceedings of the 1998 ACM/SIGDA sixth international symposium on Field programmable gate arrays (pp. 191-200).

[11] Bagalkoti, A., Shirol, S.B., Kumar, P. and BS, R., 2019, February. Design and implementation of 8-bit LFSR, bit-swapping LFSR and weighted random test pattern generator: a performance improvement. In 2019 International Conference on Intelligent Sustainable Systems (ICISS) (pp. 82-86). IEEE.

[12] Akhter, S., Saini, V. and Saini, J., 2017, February. Analysis of vedic multiplier using various adder topologies. In 2017 4th International Conference on Signal Processing and Integrated Networks (SPIN) (pp. 173-176). IEEE.

[13] D. Bankman, L. Yang, B. Moons, M. Verhelst and B. Murmann, "An Always-On 3.8 μ J/86% CIFAR-10 Mixed-Signal Binary CNN Processor With All Memory on Chip in 28-nm CMOS," in IEEE Journal of Solid-State Circuits, vol. 54, no. 1, pp. 158-172, Jan. 2019
