# Supplementary material for: Spintronic Bayesian Hardware Driven by Stochastic Magnetic Domain Wall Dynamics
Source: Adv Sci (Weinh). 2026 Mar 17;13(27):e20717. doi: 10.1002/advs.202520717 (PMC13170188; doi:10.1002/advs.202520717)
Supplement: Supplementary file 2 — Supporting File 2: advs74655‐sup‐0002‐SuppMat.docx.[Correction added on 24 March 2026, after first online publication: the Supporting Information file has been replaced in this version.] [file ADVS-13-e20717-s001.docx]

**Spintronic Bayesian Hardware Driven by Stochastic Magnetic Domain Wall Dynamics**

**Authors**

Tianyi Wang^1,*,†^, Bingqian Dai^1,*,†^, Shijie Xu^2,*^, Kin Wong^1^, Yaochen Li^1^, Yang Cheng^1^, Qingyuan Shu^1^, Haoran He^1^, Puyang Huang^1^, Hanshen Huang^1^, Xixiang Zhang^2^ and Kang L. Wang^1,†^

**Affiliations**

*^1^Department of Electrical and Computer Engineering, University of California, Los Angeles, California 90095, United States*

*^2^Physical Science and Engineering Division, King Abdullah University of Science and Technology (KAUST), Thuwal, Saudi Arabia.*

*These authors contributed equally to this work.

Corresponding author E-mail: ^†^tianyiwang0220@g.ucla.edu, ^†^bdai@g.ucla.edu, ^†^wang@ee.ucla.edu

|  |
| --- |
| **Extended Data Fig. 1 \| Mask design. (a)** Mask layout of MPC device. Spin-orbit torque (SOT) current is injected through Channels 1 and 2, which drives the DW along the magnetic stripe. The TMR signal is read out via Channels 3 and 4, which connect to the top and bottom electrodes of the MTJ respectively. DWs are initialized by Oersted currents applied through Channels 5 and 6, alternatively through Channels 7 and 8. **(b)** Detailed view of the MPC device. The grey layer represents the DW channel for DW motion. The red layer illustrates the bottom Ta contact layer, connecting the free layer of the MTJ. The black layer outlines the MTJ area, highlighting the location of MTJs. **(c)** Illustration of fabricated device and measurement setup. SOT current is injected in the bottom Ta layer. The TMR signal is measured across the MTJ stack by applying a vertical reading voltage. **(d)** Cross-section in the x-z plane of the fabricated device. **(e)** Cross-section in the y-z plane of the fabricated device. |

|  |
| --- |
| **Extended Data Fig. 2 \| Device fabrication. (a)** Simplified MTJ stack. **(b)** Photolithography and etching for device shape. **(c)** Photolithography and etching for MTJ pillar. **(d)** Photolithography and evaporation for Au/Cr MTJ Bottom electrodes. **(e)** AlO_x_ deposition by Atomic Layer Deposition (ALD). **(f)** Photolithography, etching, and evaporator for Au/Cr MTJ top electrode. |

|  |
| --- |
| **Extended Data Fig. 3 \| Mechanism of domain creation and DW motion. (a)** Schematic of domain generation. The yellow tube represents the conducting stripline, while the green arrows indicate the Oersted field generated by the current. Current pulses induce the Oersted field, which facilitates the nucleation of magnetic domains. **(b)** Schematic of DW motion. Yellow arrow represents the SOT current. |

|  |
| --- |
| **Extended Data Fig. 4 \| Gaussian distribution analysis. (a)** Quantile–Quantile plot validating that the data generated by Python follows an ideal Gaussian distribution. **(b)** Quantile-Quantile plot evaluating the Gaussian distribution of data produced by the MPC device. The data closely aligns with the reference line, indicating an excellent Gaussian fit. |

|  |
| --- |
| **Extended Data Fig. 5 \| Micromagnetic simulation for high PMA and low PMA case comparison. (a)** DW time evolution images exhibit fluctuation under thermal excitation in the high perpendicular magnetic anisotropy (PMA) state. The DW remains stable with minimal tilting due to strong PMA. **(b)** DW exhibits increased tilting under thermal excitation in the low PMA state, indicating reduced stability. |
